# Active Redesign of a Medicaid Care Management Strategy for Greater Return on Investment: Predicting Impactability

**DOI:** 10.1089/pop.2017.0122

**Published:** 2018-04-01

**Authors:** C. Annette DuBard, Carlos T. Jackson

**Affiliations:** Community Care of North Carolina, Raleigh, North Carolina.

**Keywords:** care management, predictive modeling, impactibility, accountable care, Medicaid

## Abstract

Care management of high-cost/high-needs patients is an increasingly common strategy to reduce health care costs. A variety of targeting methodologies have emerged to identify patients with high historical or predicted health care utilization, but the more pertinent question for program planners is how to identify those who are most likely to benefit from care management intervention. This paper describes the evolution of complex care management targeting strategies in Community Care of North Carolina's (CCNC) work with the statewide non-dual Medicaid population, culminating in the development of an “Impactability Score” that uses administrative data to predict achievable savings. It describes CCNC's pragmatic approach for estimating intervention effects in a historical cohort of 23,455 individuals, using a control population of 14,839 to determine expected spending at an individual level, against which actual spending could be compared. The actual-to-expected spending difference was then used as the dependent variable in a multivariate model to determine the predictive contribution of a multitude of demographic, clinical, and utilization characteristics. The coefficients from this model yielded the information required to build predictive models for prospective use. Model variables related to medication adherence and historical utilization unexplained by disease burden proved to be more important predictors of impactability than any given diagnosis or event, disease profile, or overall costs of care. Comparison of this approach to alternative targeting strategies (emergency department super-utilizers, inpatient super-utilizers, or patients with highest Hierarchical Condition Category risk scores) suggests a 2- to 3-fold higher return on investment using impactability-based targeting.

## Introduction

Care management of patients with complex care needs has become an important strategy of payers, employers, government agencies, health systems and provider groups who strive to improve outcomes and lower costs of care. Evidence of the effectiveness of care management has been mixed, with a growing literature that suggests that program savings can only be achieved through selective targeting of high-risk patients.^1–4^ Most commonly, programs target patients for care management based on specific patterns of care (such as high emergency department [ED] or inpatient utilization); by referrals from providers or other community partners; by the presence of chronic conditions or other risk factors associated with high preventable costs; or by community (underserved areas).^5–10^ Technological advances in big data management and analytic capabilities have offered further promise, with the emergence of a multitude of predictive modeling products on the market for early identification of high-cost/high-needs patients.^11,12^

Community Care of North Carolina (CCNC) has provided care management support for North Carolina Medicaid recipients for more than 2 decades, through a statewide system of locally embedded, multidisciplinary care teams supporting more than 1600 participating primary care medical homes and community partners. With a member population of more than 1.6 million individuals and the resources to provide intensive care management support to fewer than 1% of members at any given time, care management outreach and intervention must be judiciously allocated within available budget. Through rigorous evaluation of care management interventions over time, and the discipline of continuous quality improvement, CCNC has actively evolved its care management targeting strategy to better identify patients most likely to benefit from this support, moving away from a focus on “high risk” to a focus on “highly impactable.”

This paper first aims to describe CCNC's approach to estimating the impact of real-world care management interventions through pragmatic program evaluation methods, allowing for the differentiation of high-risk patients for whom care management had little-to-no impact on future outcomes, as well as lower cost and less clinically complex patients who benefit substantially from care management. It then describes the prospective application of these insights: a fundamentally different predictive modeling strategy that aims to predict “impactability”—the expected dollar savings achievable through care management intervention—rather than predicting future costs or events. Finally, this study retrospectively examines the overall savings impact of an impactability-based care management targeting strategy compared to other more common strategies such as the targeting of highest ED utilizers, highest inpatient utilizers, or patients with the highest Hierarchical Condition Category (HCC) risk scores.

## Background

CCNC's complex care management (CCM) program began in 2009, under state legislative mandate to expand community-based care coordination services beyond children and families to include aged, blind, and disabled Medicaid recipients. Initially, patients were identified for CCM in a variety of ways, most commonly by referral from the primary care provider or hospital. Claims-based reports identified additional priority patients through a clinical algorithm that initially assigned points based on a number of risk factors, including: top cost percentile; presence of diabetes, asthma, heart failure, or chronic obstructive pulmonary disease (COPD); presence of multiple chronic conditions; behavioral health comorbidity; 3 or more outpatient providers; 8 or more medications, and count of prior ED and inpatient visits. This scoring system was phased out when an indicator of above-expected costs related to potentially preventable hospital visits was introduced in 2011, and additional indicators of predicted 12-month risk of hospitalization and predicted risk of drug therapy problems were introduced in 2013. These indicators, intended to identify patients likely to benefit from care management to inform outreach priorities, were available to local care management teams within a web-based Care Management Information System and in “priority patient list” reports updated quarterly. CCNC's evaluation methodologies for estimating the savings impact of care management, isolating predictive factors, and prospectively applying those learnings for care management targeting strategy have been iteratively developed and refined since 2014. The first impactability-based scoring system for CCM prioritization was deployed in August 2015, and the current impactability model that will be described in this paper was deployed in August 2016.

Upon engagement of patients identified as priority for care management outreach, CCNC care managers comprehensively assess health status, knowledge, and behaviors; gaps in care; self-management capabilities and support network; social and financial barriers; and the goals of the patient. Care management interventions are individualized to the needs of the patient, but commonly involve medication review and reconciliation, facilitating communication with the primary care provider and specialists, motivational interviewing, health coaching and patient/caregiver education, and linkage to community resources.^13^ The CCM program operates within a fixed budget under capitated management fees from the state Medicaid agency,^14,15^ such that demand for care management services has consistently exceeded capacity.

## Methods

### Data sources

Evaluation of the effectiveness of the CCM program and subsequent development and testing of CCNC's impactability-based predictive models relied on statewide North Carolina administrative data for the time period January 1, 2010, through May 1, 2017. Data included eligibility and enrollment files, all medical and pharmacy claims paid by Medicaid, and encounter claims from all managed care organizations administering carve-out behavioral health benefits. Care management interventions were electronically documented in standardized format in CCNC's web-based care management information system (CMIS). CMIS also captures structured, standardized comprehensive health assessment and social determinant data ascertained by the care manager. These data are used here to describe the characteristics of patients engaged in CCM, but were not used in the development of the impactability models as the goal was to develop a screening approach using only administrative data available for the whole population.

Patient disease burden was characterized using the categorical and hierarchical Clinical Risk Group (CRG) methodology developed by 3M Health Information Systems (Salt Lake City, UT), which assigns individuals to one of 1075 mutually exclusive groups characterized by number and type of chronic conditions and associated severity.^16^ Each beneficiary was assigned a CRG risk score that reflected average total costs of care within that CRG relative to the CCNC population as a whole. 3M Health Information Systems' Population-Focused Preventables Software was used to identify potentially preventable admissions, readmissions, and ED visits.

### Model variables

For the linear regression models used to estimate care management effects and predictive models used to estimate achievable savings, independent variables included: age, sex, race, ethnicity, disability status, foster care status, ED visit count, inpatient visit count, CRG weight, presence of specific chronic conditions, number of chronic conditions, number of chronic medications filled, number of acute medications filled, and total cost of care. “Above-expected potentially preventable costs” (AEPPC) is a derived variable that considers only costs related to potentially preventable admissions, readmissions, and ED visits. The AEPPC for an individual is the difference between actual potentially preventable costs and the median of potentially preventable costs among CCNC-enrolled beneficiaries in the same CRG over a 12-month period. Additional derived variables included monthly spending trajectory over the most recent 12-month periods, and 2 indicators of adherence to chronic medications: (1) proportion of days covered within selected therapeutic classes, and (2) gaps in fills for selected therapeutic classes. Two- and 3-level interactions were tested and used when they added significant predictive value.

### Study population

The authors identified 23,455 non-dual, continuously eligible, CCNC-enrolled Medicaid beneficiaries who received some level of care management between October 2011 and September 2012, and had at least 1 potentially preventable admission, readmission, or ED visit in the year prior to initiation of care management. Control subjects were selected from a historical period, January-December 2010, during which CCNC's CCM program was not yet fully to scale. Control subjects were a sample of 14,839 continuously eligible, CCNC-enrolled Medicaid beneficiaries who had at least 1 potentially preventable admission, readmission, or ED visit during the year but were not approached for care management during that year or through 6 months of follow-up, to June 2011.

To mitigate against unmeasurable selection bias, patients were considered to have received care management if they had at least 1 direct encounter with a care manager by phone or face-to-face. This low threshold for inclusion in the intervention group demonstrated intent-to-treat for care management regardless of the patient's subsequent willingness to engage. The actual components and duration of care management intervention varied widely across subjects, according to the needs of the individual. For measurement of total costs of care, the pre period for patients in the intervention group was defined as the 6 months prior to the initiation of care management, and the post period was the 6 months after the intervention began. For control subjects, January 1, 2011 was considered the start date for pre–post evaluation.

### Development of impactability scores

To estimate the savings impact attributable to care management intervention at the individual patient level, the authors compared the pre–post difference in total cost of care for intervention patients to the pre–post spending difference that would have been expected for that patient in the absence of intervention. To calculate this “expected” value of spending difference, the authors first conducted a linear regression analysis using data from control patients only. The dependent variable was the post period spend, and the independent variables included a multitude of patient demographic, clinical, cost, and hospital utilization characteristics from the pre period. The resulting coefficients provided an estimate of predicted influence of each of these independent variables on post period spend in the absence of intervention. By applying these coefficients from the control group model to patients in the intervention group, using the variables from the intervention patient's pre period as the independent variables, “expected” costs in the post period could then be predicted for patients in the intervention group. Then, for each individual patient, the authors calculated the difference between their expected spend in the post period and their actual spend in the post period and created a new measure of “variance from expected.”

Finally, to be able to prospectively estimate impactability, a second linear regression analysis was conducted, using “variance from expected” as the dependent variable, with the same pre period data for the intervention group as independent variables. This final model produced the coefficients necessary to derive impactability scores for prospective use. When applied to known variables in a current cohort of patients, the model returns a number value that represents the predicted variance from expected in per member per month (PMPM) spending over 6 months. Multiplying this “Impactability Score” by 6 provides an estimate of total gross savings that can be expected, on average, from care management intervention.

### Comparison of impactability-based targeting to other approaches

To further validate this targeting methodology, the authors returned to the study population of 23,455 intervention patients and 14,830 control patients described, and identified the top 5000 members based on predicted impactability and each of 4 alternative strategies: highest ED utilizers, highest inpatient utilizers, anyone with a prior potentially preventable inpatient or ED visit (random sample of 5000), and highest HCC risk score. HCC is a standard risk-adjustment score utilized by the Centers for Medicare & Medicaid Services; higher prospective HCC scores reflect proportionally higher expected future costs based on an individual's current medical conditions. Baseline year utilization determined assignment into the 4 study groups. For intervention subjects, the baseline year was the year prior to each individual's care management intervention start date. For control subjects, the baseline year was calendar year 2010. Using 6-month pre–post, difference-in-difference analysis, the authors compared total cost of care trends for patients who received care management vs. control patients within each cohort.

For measurement of total costs of care, the pre period for patients in the intervention group was defined as the 6 months prior to the initiation of care management, and the post period was the 6 months after the intervention began. For control subjects, January 1, 2011 was considered the start date for pre–post evaluation. As before, patients were considered to have received care management if they had, at a minimum, a direct encounter between the care manager and the patient that was either by phone or face-to-face. Subjects in the control groups were similar to subjects in the intervention groups with the exception that they were not approached for care management. The difference-in-difference analysis helps to control for unmeasured external factors that may influence spending trends.

In the absence of a randomized controlled trial, the possibility of selection bias remains, but any remaining biases should be similar across the 4 study groups, preserving the ability to draw conclusions about relative effects. For each of the targeting approaches evaluated, the authors compared the difference in total spend PMPM in the post period minus the total spend PMPM in the pre period to calculate a change score for each individual. Because this had a non-normal distribution, intervention effects were evaluated using the Mann-Whitney U-Test, a nonparametric test that treats the cost differences as rank-ordered data. The resulting statistic is based on a *z* distribution.

## Results

### Characteristics of highly impactable patients

**FIG. 1.**
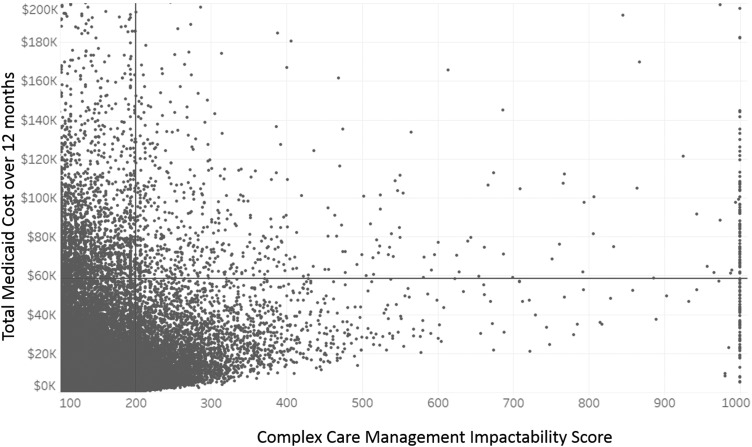
Relationship between complex care management impactability scores and total Medicaid spend. These data reflect 1,753,407 non-dual North Carolina Medicaid recipients as of May 2017. Data truncated at <100 and >1000 for the x-axis, and at >$200,000 for the y-axis, for ease of interpretability. Individuals to the right of the *vertical bar* have an Impactability Score >200, the threshold that Community Care of North Carolina currently uses to prioritize care team outreach. The *horizontal bar* represents the equivalent number of patients on the cost scale: if prioritization were based strictly on high-cost/high-needs criteria, care management would be deployed to the people above that line. Such an approach would capture many high-risk patients who are not likely to benefit from care management (*left upper quadrant*), while missing many lower risk patients for whom care management is more likely to yield savings (*right lower quadrant*).

Figure 1 displays the distribution of impactability scores and total Medicaid spend for the current NC Medicaid non-dual population. As illustrated, not all high-cost patients have high impactability scores, and not all patients with high impactability scores have high costs.

Demographic characteristic, chronic disease prevalence, and historical cost and utilization patterns are described in Table 1, for North Carolina Medicaid recipients with low (0–199), medium (200–499), and high Impactability Scores. As might be expected, recipients with higher predicted probability of savings benefit from care management have substantially higher historical costs, inpatient, and ED utilization. Adult Medicaid recipients and those with chronic conditions are disproportionately represented among those with higher Impactability Scores. Mental illness was found in 55% and 48% of high- and medium- impactability groups compared to 12.6% of members with low impactability; while 3 or more chronic conditions were present in 71.6%, 38.1%, and 5.8% respectively.

**Table 1.** Characteristics of the North Carolina Medicaid Population, by Impactability Score

*Impactability Group*	*LOW (0–199)*	*MEDIUM (200–499)*	*HIGH (500–1000)*
N	1,740,530	12,515	362
Demographic Characteristics			
Age 0–20	71.8%	33.7%	14.9%
Female	56.3%	69.0%	61.1%
African American	36.6%	38.0%	38.1%
White	56.6%	57.4%	58.8%
Hispanic	15.8%	4.8%	3.0%
Chronic Conditions			
Attention-deficit/hyperactivity disorder	4.4%	7.8%	5.0%
Asthma	8.0%	28.2%	27.9%
Bipolar Disorder	1.2%	10.3%	14.1%
Cancer	0.5%	2.8%	8.8%
Congestive Heart Failure	0.2%	2.6%	13.0%
Chronic Gastrointestinal Disorder	4.2%	23.7%	47.2%
Chronic Kidney Disease	0.4%	3.1%	14.4%
Chronic Neurological Disorder	1.9%	10.8%	30.1%
Chronic Obstructive Pulmonary Disease	1.2%	8.2%	21.0%
Depression	2.6%	19.8%	28.5%
Developmental Disability	3.5%	4.5%	8.6%
Diabetes	2.4%	14.5%	37.3%
HIV	0.2%	1.1%	1.9%
Hypertension	5.1%	30.0%	58.0%
Ischemic Vacular Disease	0.9%	6.9%	23.8%
Mental Illness (any diagnosis)	12.6%	48.4%	55.0%
Schizophrenia	0.7%	3.7%	6.1%
Substance Abuse	1.4%	11.5%	24.3%
Sickle Cell Disease	0.1%	1.2%	3.6%
3 or More Chronic Conditions	5.8%	38.1%	71.6%
Health Care Utilization in Prior 12 Months			
Total costs per member per month	$315.56	$1,321.97	$5,823.72
Inpatient visits per 100 members	9.5	67.3	291.0
Emergency department visits per 100 members	55.3	493.5	1,704.0

These data reflect 1,753,407 non-dual North Carolina Medicaid recipients as of May 2017.

It is important to recognize, however, the clinical risk profile of the patient does not determine impactability in and of itself. This is illustrated in Table 2, which examines the frequency of Impactability Scores >200 within selected clinical risk groups. For example, fewer than 1% of recipients with uncomplicated asthma meet this threshold, as do fewer than 0.3% of those with major mental Illness or substance abuse diagnosis in the absence of other significant illness. Even among recipients with very high disease burden, such as those on dialysis with diabetes; or those with congestive heart failure, COPD, and additional chronic disease; the CCM Impactability model generated a score above 200 for only 11.3% and 14.6%, respectively. This is because model variables related to adherence to chronic medications, ED utilization, and historical spending unexplained by disease burden proved to be more important predictors of impactability than any given diagnosis or event, overall disease profile, or overall costs of care.

**Table 2.** Prevalence of Impactability, by Select Clinical Risk Groups

*Select Clinical Risk Groups*	*Number of Medicaid Members*	*Number with CCM Impactability Score ≥200*	*Percent with CCM Impactability Score ≥200*
Healthy	656,579	157	0.02%
Major Mental Illness or Substance Abuse Diagnosis without Other Significant Illness	11,165	31	0.28%
Asthma Level - 1	25,586	240	0.94%
Congenital Quadriplegia, Diplegia, or Hemiplegia Level - 2	1030	10	0.97%
Hypertension Level - 1	9434	129	1.37%
HIV Disease Level - 1	1348	19	1.41%
Chronic Obstructive Pulmonary Disease and Bronchiectasis Level - 1	517	8	1.55%
Diabetes Level - 1	1388	23	1.66%
Schizophrenia Level - 1	4724	111	2.35%
Epilepsy Level - 1	3898	97	2.49%
Depression Level - 1	3479	90	2.59%
Schizophrenia and Other Moderate Chronic Disease Level - 3	2169	109	5.03%
Asthma and Other Moderate Chronic Disease Level - 3	2228	126	5.66%
Diabetes and Other Moderate Chronic Disease Level - 6	1046	69	6.60%
HIV Disease Level - 3	974	86	8.83%
Chronic Obstructive Pulmonary Disease and Other Dominant Chronic Disease Level - 6	441	46	10.43%
Dialysis with Diabetes Level - 4	337	38	11.28%
Congestive Heart Failure - Chronic Obstructive Pulmonary Disease - Other Dominant Chronic Disease Level - 5	110	16	14.55%

CCM, complex care management.

Although the CCM Impactability model relies solely on historical claims data as an initial screening mechanism for care management outreach, CCNC care managers subsequently conduct a comprehensive health assessment with documentation of social, financial, environmental, and other challenges reported by patients or families engaged in care management. Table 3 describes the prevalence of social determinants among patients with Impactability Scores >200 assessed by a care manager. As illustrated, this “most impactable” population had many significant social risk factors such as mental illness, unstable support system, lack of transportation, unstable housing, substance abuse, trauma or abuse, unmet nutritional needs, and illiteracy. Approximately three quarters of the patients had at least 1 of these risk factors, and nearly half had 2 or more. These factors are known to influence health care utilization and health outcomes, and often become the focus of intervention for the care management team.

**Table 3.** Social Determinants of Health Among Impactable Patients Engaged in Complex Care Management

Social Risk Factor^*^	*N*	*%*
Mental Illness	12,322	67%
Unstable Support System	4253	23%
Lack Transportation	3943	21%
Unstable Housing	2642	14%
Substance Abuse	2289	12%
Trauma/abuse	2592	14%
Nutritional Needs	2256	12%
Illiteracy	2029	11%
ANY of the above	14,387	78%
ANY of the above (excluding Mental Illness)	9315	51%
≥2 Risk Factors	8202	44%
≥4 Risk Factors	2703	15%
Total N	18,439	100%

^*^ Based on care manager interviews with 18,439 patients and their families engaged in care management between August 2016-May 2017.

### Comparison of impactability-based targeting to other common targeting strategies

Table 4 describes the patients retroactively identified to have met various prioritization criteria using 5 alternative targeting strategies, within the retrospective study cohort of 38,294 non-dual CCNC-enrolled Medicaid beneficiaries with at least 1 inpatient or ED visit in the baseline period: the top 5000 based on CCM Impactability Score, the top 5000 ED utilizers, the top 5000 inpatient utilizers, the top 5000 based on HCC risk score, and a random sample of 5000. Although each group would be considered “high-risk” compared to the general Medicaid population, they are notably different from each other with regard to average impactability score, historical inpatient and ED utilization, and HCC score. Within each group, patients who received any degree of care management assessment or intervention were compared to those who did not. Of note, those who received the intervention had higher risk profiles than those who did not, consistent with the intent of care managers to prioritize highest-needs patients throughout the history of the program—a selection bias that would tend to underestimate intervention effects.

**Table 4.** Study Population for Estimation of Care Management Savings by Targeting Strategy

*Targeting Strategy*	*Received Care Management*	*N*	*Mean Impactability Score*	*Mean ED Visits in Prior Year*	*Mean IP Admits in Prior Year*	*Mean HCC Score*
Top CCM Impactability Scores	NO	1525	388	7.4	1.4	1.8
	YES	3475	555	6.9	1.0	1.6
	Total	5000	504	7.1	1.1	1.7
Top ED Super-utilizers	NO	1356	266	12.8	1.0	1.4
	YES	3644	217	14.2	1.0	1.5
	Total	5000	230	13.8	1.0	1.4
Top IP Super-utilizers	NO	1638	263	5.4	2.5	2.1
	YES	3362	231	6.6	2.8	2.3
	Total	5000	241	6.2	2.7	2.2
Top HCC Scores	NO	1587	258	5.7	1.7	2.8
	YES	3413	252	7.4	1.9	2.9
	Total	5000	254	6.9	1.9	2.9
Random	NO	1031	152	2.5	0.3	0.6
	YES	3969	145	2.8	0.4	0.6
	Total	5000	146	2.8	0.4	0.6

CCM, complex care management; ED, emergency department; HCC, Hierarchical Condition Category; IP, inpatient.

For all 5 targeting strategies, patients who received care management experienced a reduction in spend that was greater than the change in spend observed in their respective comparison groups (Table 5). Inpatient super-utilizers and those with highest HCC scores had the highest baseline spending, but downward spending trends were notable even for those who did not receive care management, reflecting a natural regression to the mean. Difference-in-difference analysis estimated a care management savings impact of $5,922 per patient over the 6-month follow-up period for patients with the highest CCM Impactability Scores, compared to $2,748 for ED super-utilizers, $2,178 for inpatient super-utilizers, $1,650 for highest HCC scores, and $1,470 for patients with any prior inpatient or ED use.

**Table 5.** Estimated Savings Attributable to Care Management by Targeting Strategy

*Received Complex Care Management*	*N*	*Total Spend PMPM (PRE)*	*Total Spend PMPM (POST)*	*Total Spend PMPM (DIFF)*	*Net Difference (Estimated Impact of Care Management)*	*Mann Whitney U Test (z-statistic)*
Top Scoring Patients (Complex Care Management Impactability Score)						
NO	1525	$2474	$2368	−$106		
YES	3475	$2848	$1756	−$1,093	**-$987**	-13.52^*^
Top ED “Super-utilizers”						
NO	1356	$2000	$1894	−$105		
YES	3644	$2547	$1984	−$563	**-$458**	-5.37^*^
Top Inpatient “Super-utilizers”						
NO	1638	$2989	$2610	−$379		
YES	3362	$4024	$3282	−$742	**-$363**	**-0.87**
Top HCC Scores						
NO	1587	$3290	$2967	−$323		
YES	3413	$3867	$3270	−$597	**-$274**	-3.53^*^
Random						
NO	1031	$732	$715	−$17		
YES	3969	$1095	$833	−$262	**-$245**	-5.26^*^

^*^ *P* < 0.001

ED, emergency department; HCC, Hierarchical Condition Category; PMPM, per member per month.

## Discussion

As payers, providers, and other health care stakeholders pursue cost containment through care management of high-cost/high-needs patients, there is a great need for pragmatic approaches to real-world program evaluation, and a stronger body of evidence to guide targeting and intervention strategies. CCNC's active redesign of its CCM strategy over the past decade has been driven by a commitment to continuous quality improvement, relying on creative but conservative approaches to estimate intervention effects and applying those learnings in iterative fashion. Undoubtedly this commitment has contributed to CCNC's renowned success in reducing hospital inpatient and ED utilization, and total costs of care, among NC Medicaid beneficiaries.^17–20^ CCNC's novel approach for generating patient-specific estimates of savings attributable to care management, in order to fine-tune models for predicting this “change from expected” – or “impactability” – should be a helpful framework for others who seek to prioritize care management resource allocation in a manner that maximizes population impact and return on investment.

Importantly, CCNC's experience reveals that “high-cost/high-needs” is not the same thing as “highly impactable.” Targeting strategies that seek to identify patients based on high current or predicted costs or utilization are likely to identify large numbers of individuals whose health care needs will not be meaningfully altered by care management intervention. Conversely, many individuals at the lower end of the cost spectrum may benefit substantially. CCNC has demonstrated that reliable indicators of impactability can be gleaned from historical administrative data alone, and that patterns such as potentially preventable health care utilization that is outside the norm for an individual's disease burden, and prescription fill adherence rates for certain chronic medications, are stronger predictors of responsiveness to care management than the presence of a high-risk diagnosis, super-utilizer status, or having high historical or predicted costs. These analyses suggest that the return on investment from care management intervention is 2- to 3-fold higher with impactability-based targeting compared to these more common approaches.

Impactability-based targeting using administrative data alone provides an efficient first-pass screening mechanism, to allow for more focused outreach by care management teams to the small percentage of the population most likely to benefit. Administrative data do not include many important indicators relevant to an individual's care management needs, particularly related to social factors that are recognized to have a greater impact on health outcomes than medical care.^21^ These social determinants are systematically identified by CCNC care managers as part of the assessment and care planning process, are found at very high rates among patients with high Impactability Scores from the claims-based screening, and often become a focus of intervention efforts. This suggests that the influential variables in the CCM Impactability model may be serving as markers of more upstream drivers of disease exacerbation and health care costs, such as lack of social support, food or housing insecurity, and low health literacy.

The goal of CCNC's Impactability-based targeting is to find individuals with higher probabilities of benefitting from care management intervention, specifically in terms of near-term cost savings. Because the scores reflect probability, the savings estimates are not precise at the level of the individual: some individuals will benefit more and others less than the score suggests. Expressing the model output as a score that reflects expected gross dollar savings is most accurate at an aggregated level, providing important utility for program planning. The threshold score for determining care management priority can be titrated to local circumstances, acknowledging that the cost of care management intervention can be quite variable depending on factors such as the robustness of existing administrative infrastructure, salary norms, and travel time in rural vs. urban settings. Program planners will have a reasonable sense of achievable net savings taking local cost structures into account, and can use Impactability Score distribution across the population to determine the optimal investment in care management to yield maximal savings, or to assure maximal savings within a fixed budget.

It also should be acknowledged that cost savings is not the only potential benefit of care management, and many may argue that improving patient or provider experience, or improving patient outcomes over a longer time horizon, is of merit whether or not savings accrue in the near term.^22^ Future research must continue to rigorously delineate the potential for care management to impact all of these domains—what works and what doesn't, when, for whom? Alternative predictive modeling techniques, including cluster analysis and other machine learning approaches, may provide additional insights. To optimize the benefits of care management within the realities of resource constraints, future advances in predictive analytic strategies will require a shift in focus from risk to impactability.
